# Rebuilding of Turbocharger Shafts by Hardfacing

**DOI:** 10.3390/ma15165761

**Published:** 2022-08-20

**Authors:** Bogdan Kupiec, Zenon Opiekun, Andrzej Dec

**Affiliations:** Department of Casting and Welding, Faculty of Mechanical Engineering and Aeronautics, Rzeszow University of Technology, Al. Powstańców Warszawy 12, 35-959 Rzeszów, Poland

**Keywords:** GTAW hardfacing, flux-cored wire, turbocharger shaft

## Abstract

This paper presents the results of structural tests and hardness measurements of rebuilding coatings manually applied by the gas tungsten arc welding (GTAW) method on damaged surfaces of steel shafts of turbochargers of automotive engines. Single- and double-layer coatings were applied in an argon atmosphere by fusing a 1.2 mm diameter wire with a Fluxofil M58 flux core, using a current of 35A and an arc voltage of 8–9 V. The hardfacing resulted in coatings with a martensitic–bainitic structure with fine dispersed carbides rich in M23C6-type chromium. The hardness of the coatings on the rebuilt shafts averaged from about 740HV5 for the single-layer coating to about 770HV5 for the double-layer coating and was two times higher than the hardness of the tempered shafts without coatings.

## 1. Introduction

**Figure 1:**
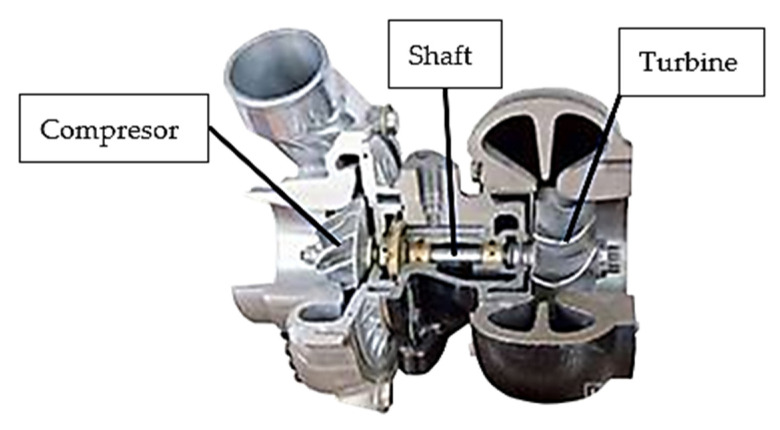
Components of a turbocharger.

Common devices used to increase the specific power of internal combustion engines are turbochargers. The air turbochargers installed as standard in car engines have a design consisting of three main parts: the compressor, where the air is compressed by a skewed-blade rotor, the centrifugal (radial-axial) turbine, where the exhaust gas is expanded, and the turbocharger shaft (Figure 1) [1,2,3].

The construction of a turbocharger for a motor vehicle is not very complicated, yet the operating conditions require that its components are carefully manufactured, continuously and properly lubricated, and also cooled.

Operational damage to turbochargers is caused by excessively high rotor speeds, which significantly increase the centrifugal forces acting on their components, excessively high temperatures of the gases supplied to the turbine, which can induce dimensional change processes caused by creep, and the deposition of thermolysis products of the superheated oil used to lubricate the shaft [4,5,6].

The gases flowing through the turbocharger should not contain solid impurities that could cause damage to its moving components, especially the shafts [7]. Turbocharger shafts are made from low-alloy structural steel for tempering [8], as well as corrosion-resistant chrome-nickel steel [9,10]. The shafts are permanently connected to the turbine rotor by welding. The temperature of the exhaust gas flowing into the turbocharger should not exceed about 800 °C for a compression-ignition engine and about 1000 °C for a spark-ignition engine. Higher exhaust gas temperatures can cause damage to the turbocharger through accelerated corrosion, deformation of its body, and thermal fatigue of the shafts [11].

**Figure 2:**
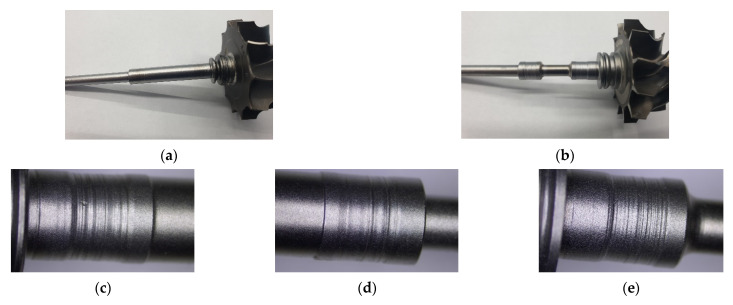
Views of the shafts with turbocharger turbine rotors. Renault Laguna 1.9 DCI, Garret 708,639 model (**a**); Peugeot 3071.6HDI, Garret 7534200375J6 model (**b**). Worn shaft journal surfaces (examples) (**c**–**e**).

The journals of turbocharger shafts wear abrasively in the areas where the components work together (at the kinetic nodes of the shaft—plain bearing) [12]. The loose particles of hard abrasive material [13,14], during the relative movement of the friction surfaces, cause damage and destruction to the shaft journals by micro-cutting, scratching, furrowing, abrading, and tearing off the protrusions of bumps (Figure 2).

Damage to the kinematic nodes of turbochargers causes characteristic vibro-acoustic symptoms and the reduced performance of the internal combustion engine [15,16,17,18,19,20]. With signs of wear and tear, the shafts with turbine rotors are replaced in their entirety, even after the turbocharger has been in operation for a short time.

The aim of this article is to present the possibility of rebuilding worn top layers of turbocharger shafts by the arc welding of wear-resistant and hard coatings. The turbocharger shaft was regenerated using the GTAW method.

The GTAW method is one of the welding methods for producing welded joints and overlaying coatings, mainly of special steels and non-ferrous metal alloys. The GTAW method is one of the cleanest welding methods in terms of metallurgy. Welded joints and padding welds made with this method are characterized by very high purity and are free from defects. The applied shielding and plasma-forming gases, such as argon and helium or their mixtures, favor a very good shielding of the surfacing area with various coating materials. In the case of surfacing coatings on steel elements with wire with a powder core, the GTAW method gives one of the best coatings. Argon of purity 4.6 (99.996%) as an inert gas of a higher density (45% higher density than air) protects very well, preventing oxidation during surfacing [21,22].

## 2. Research Material and Methodology

**Figure 3:**
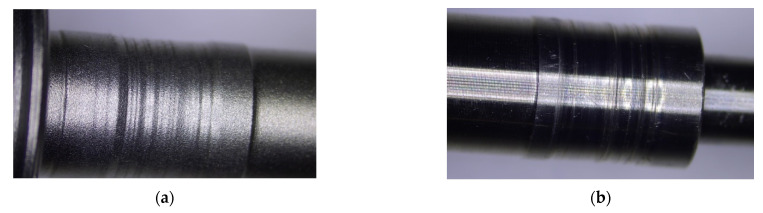
Worn top layers of turbocharger shaft journals: Renault Laguna 1.9 DCI, Garret 708,639 model (**a**), Peugeot 3071.6HDI, Garret 7534200375J6 model (**b**).

Two shafts with turbine rotors taken from the turbochargers of the Peugeot 307-1.6 HDI and Renault Laguna 1.9 DCI cars were adopted for rebuilding by hardfacing. The top layers of the journals of these shafts were severely damaged by their abrasive wear in the areas of interaction: journal-plain bearing. The thickness of the unevenly worn top layers of the journals averaged 0.2–0.3 mm. Compared to the journal diameters of new shafts of an, approximately, 7.8 mm diameter, the loss in thickness represents 4–8% of the diameter. The unevenly worn top layers of the shaft journals mainly showed deep scratches and furrowing effects caused by displaced loose particles of hard abrasive (Figure 3).

**Figure 4:**
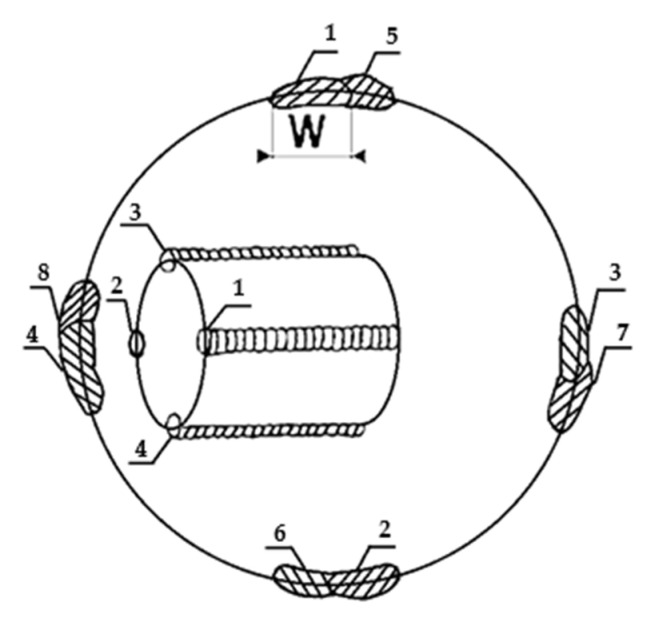
Laying sequence of the individual beads of the hardfacing coating (diagram). The numbers 1–4 in the internal diagram show the order in which the first stitches are laid on the damaged turbocharger shaft. The numbers 1–8 on the outer circle show the execution of the next stitches.

The rebuilding of the top layers of the shaft journals was carried out by hardfacing them with the GTAW method using argon as a shielding and plasma-forming gas at a flow rate of approximately 8 L/min, using a wire with a diameter of 1.2 mm and a flux core containing approximately 0.6% C, 1.9% Mn, 0.7% Si, 5.4% Cr, and the remainder being Fe. The hardfacing of the coatings was carried out manually using a current of about 35A and an arc voltage of 8–9 V. In order to cover the entire surface of the shaft journal, beads approximately 2.5 mm wide were laid lengthwise so that they overlapped each other by approximately 0.5 of their width (W), according to the diagram shown in Figure 4.

**Figure 5:**
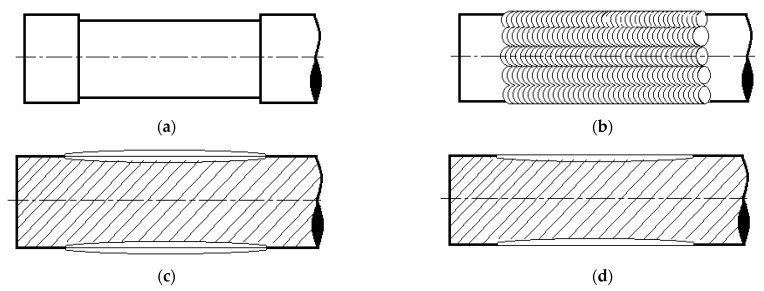
Course of rebuilding by hardfacing of shaft journals. Sandblasted surface of used shaft (**a**), bead pattern after coating (**b**,**c**), shaft after finish grinding (**d**) (diagram).

The systematic arrangement of beads around the periphery of the shaft journal ensures that axiality is maintained despite the small diameter of the shaft. Similar to the single-layer coating, a two-layer coating was made on the shaft journal by longitudinally hardfacing the second layer of the coating, respecting the width of the beads and the order in which they were laid. The course of the hardfacing of the turbocharger shaft journals is shown in Figure 5.

Metallographic examination of the shafts was carried out on metallographic specimens, on their cross-sections transverse to the direction of hardfacing of the coatings. The metallographic specimens were made by mechanical polishing and etching of the cross-sections of the shafts embedded in an electrically conductive mass. The macrostructure was observed using a Makrolite optical microscope, and the microstructure using a Neophot 2 metallographic light microscope and a Tescan scanning electron microscope with an Oxford EDS chemical composition microanalysis attachment. Shaft hardness and coating hardness were measured using a ZHV10 hardness tester, Zwick/Roell.

The chemical composition of the steel from which the turbocharger shafts were made was determined by spectral analysis using a Bruker Q4 Tasmann spectrometer.

## 3. Findings and Discussion

### 3.1. Metallographic Examination

**Figure 6:**
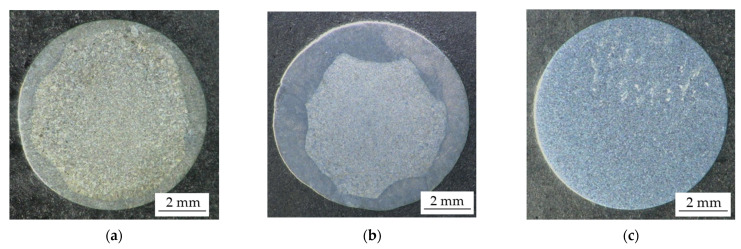
Cross-sections of turbocharger shaft journals. Single-layer coating (**a**), double-layer coating (**b**), new shaft journal (**c**). The specimens were etched with Kalling’s reagent; 3× magnification.

The macro-structures of the shaft journal cross-sections after hardfacing of the single-layer and double-layer coating, as well as the cross-section of the new shaft (without hardfacing), are shown in Figure 6.

The effect of the arc manual longitudinal hardfacing of the journals is uneven coating thicknesses, with the coating made by double-layer hardfacing having the greater average thickness. In both cases of single- and double-layer hardfacing, complete coverage of the journal surfaces with flux-cored wire hardfacing coatings was achieved (after finishing machining).

**Figure 7:**
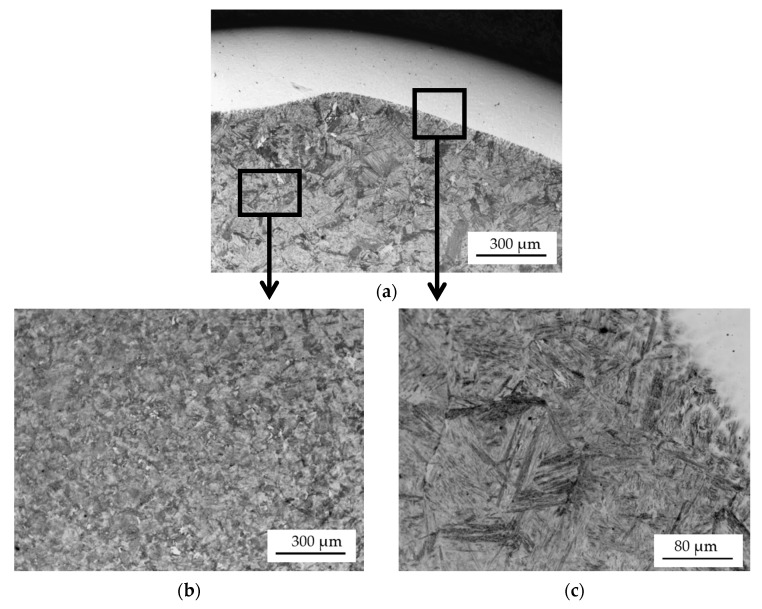
Microstructure of a turbocharger shaft journal cross-section after single-layer hardfacing: bright padding weld coating (**a**), shaft journal core (**b**), transition zone: coating—shaft journal material (**c**). The specimen was etched with 4% nital.

**Figure 8:**
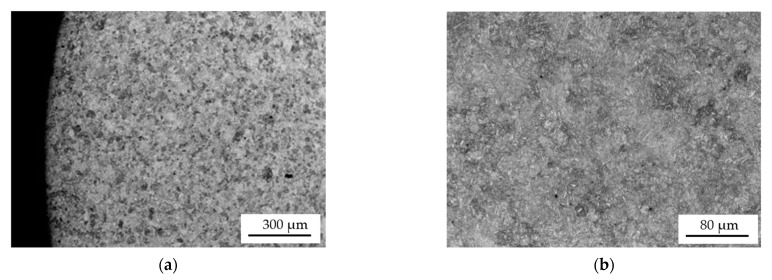
Microstructure of the new shaft journal (without coating). The specimen was etched with Kalling’s reagent (**a**,**b**).

**Figure 9:**
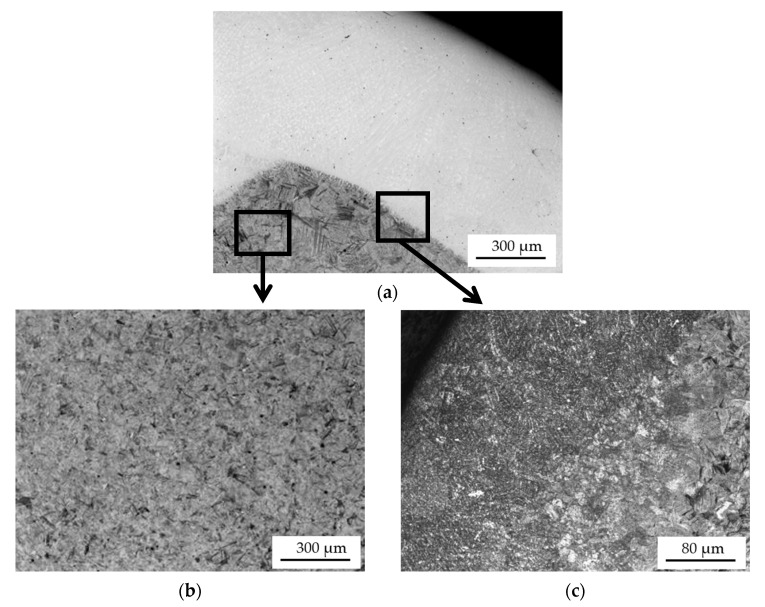
Microstructure of a cross-section of a turbocharger shaft journal after two-layer hardfacing: bright padding weld coating (etched with 4% nital) (**a**), microstructure of the shaft journal core (**b**), transition zone: coating—material (etched with Kalling’s reagent) (**c**).

The microstructures of the shaft journals observed using a light metallographic microscope are shown in Figure 7, Figure 8 and Figure 9.

The microstructure of the two coatings arc-applied with a flux-cored wire is similar. Their microstructure, revealed by etching with Kalling’s reagent (Figure 9c), is fine-grained. In contrast, the sub-coating zone and core of the single-layer hardfaced shaft is coarse-grained. The 100–150 µm grains are coarse-grained tempered martensite. In the case of the double-layer coating, by reheating the core of the shaft journal during the application of the second coating, its structure was crystallized and it has a fine-grained structure similar to that of the new shaft journal (Figure 8a and Figure 9b).

**Figure 10:**
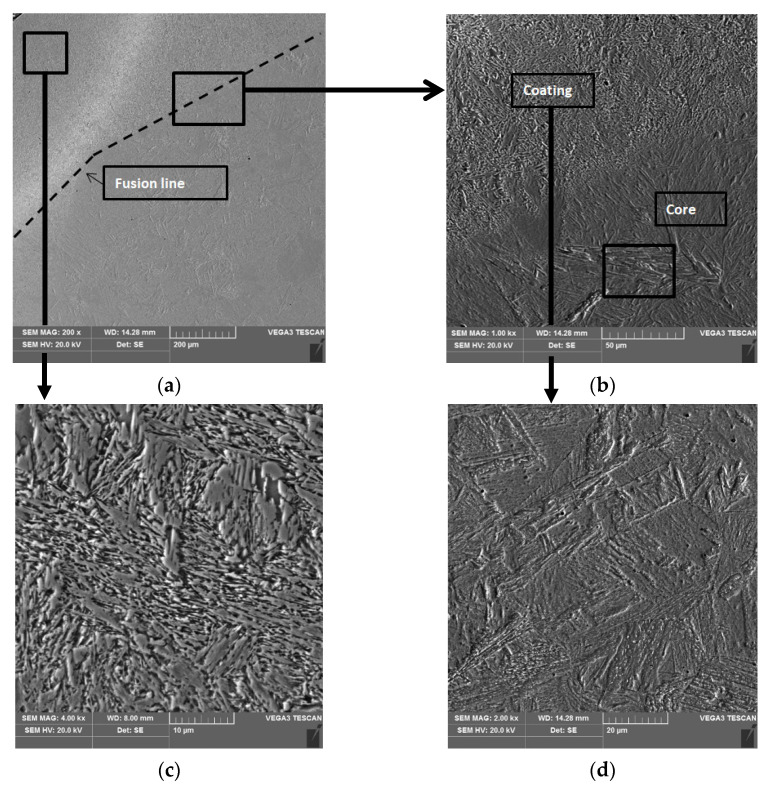
Microstructure of a turbocharger shaft journal cross-section after single-layer hardfacing. Transition zone microstructure, coating-core (**a**), detail from (**a**), coating-core (**b**), coating microstructure (**c**), core microstructure (**d**).

**Figure 11:**
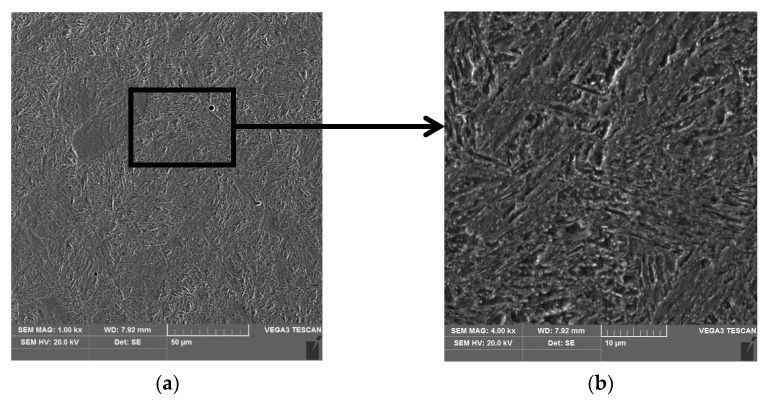
Microstructure of the new shaft journal (without hardfacing) (**a**), detail from (**a**) visible fine grains of highly tempered martensite (**b**).

**Figure 12:**
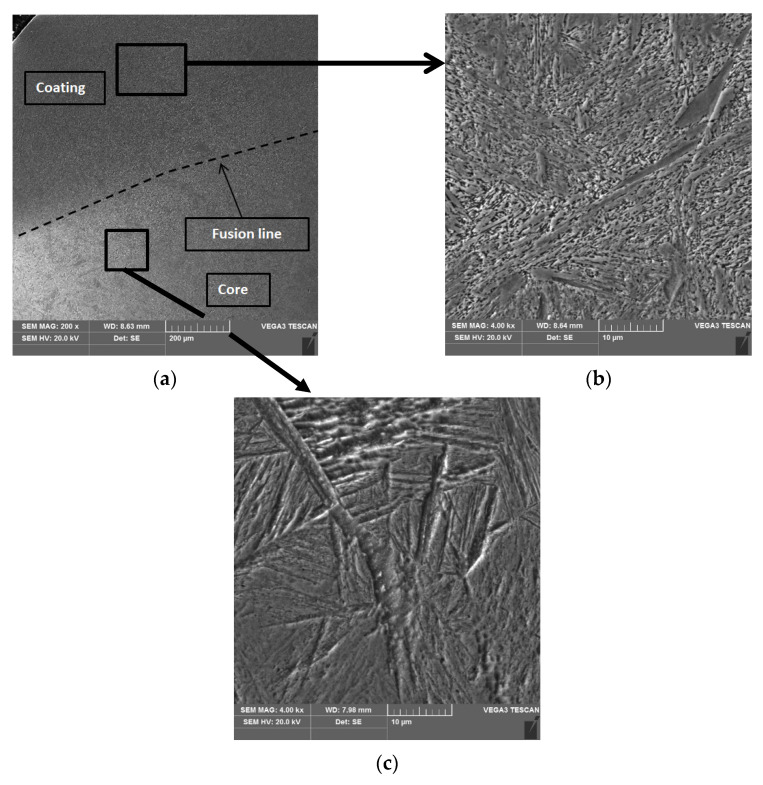
Microstructure of a turbocharger shaft journal cross-section after double-layer hardfacing. Transition zone microstructure, coating-core (**a**), coating microstructure (**b**), core microstructure (**c**).

The results of the microstructural examination of the turbocharger shaft journals using a scanning electron microscope (SEM) with an X-ray microanalysis (EDS) attachment are shown in Figure 10, Figure 11 and Figure 12. Metallographic specimens for these studies were etched more strongly with Kalling’s reagent.

The coatings that are arc hardfaced with a flux-cored wire have a similar microstructure (Figure 10c and Figure 12b). The structure of the coatings is a fine-acicular mixture of martensite and lower bainite with dispersed carbides. The microstructure of the core of a single-layer hardfaced shaft is coarse-grained (Figure 10d) with coarse-plate martensite and lower bainite. The core of the double-layer hardfaced shaft is fine-grained.

For the turbocharger shafts, a low-alloy structural steel for tempering was used with mark 42CrMo4 (acc. to PN-EN 10083-1, 1999) and the following chemical composition; 0.44% C, 0.94% Cr, 0.17% Mo, 0.17% Si, 0.73% Mn, and the remainder being Fe. The structure of the tempered shafts is fine-grained and is composed of highly tempered martensite (Figure 11).

### 3.2. X-ray Microanalysis

**Figure 13:**
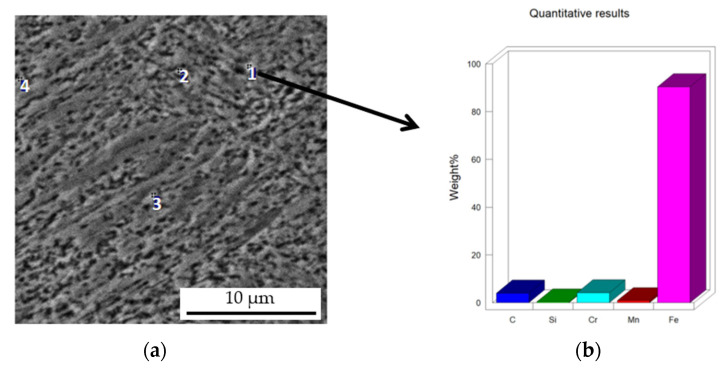
Microstructure of the flux-cored arc-hardfaced coating on the turbocharger shaft journal with micro-areas (points1–4) (**a**), bar chart of elemental content in micro-area 1 (**b**).

The place of performance of the spot X-ray microanalysis of the coating is shown in Figure 13a. The results are summarized in Table 1. Fine carbide separations, dispersively distributed between the lamellar–acicular martensite, were analyzed.

These tests confirmed that the fine spherical dispersionally distributed phases in the martensitic matrix of the hardfaced coating are iron- and chromium-containing carbides of the M_23_C_6_-type coatings. These carbides are hard, therefore, the hardfaced coating should have a higher abrasion resistance than the original uncoated rollers.

### 3.3. Hardness Measurements

Measurements of the hardness of the cores and journal coating of the turbocharger shafts were carried out using a Vickers indenter hardness tester under a 5N load. Five measurements were taken for each coating and core of the hardfaced shafts and the new shaft. Table 2 summarizes the results of these measurements.

The hardnesses of the coatings range from approx. 740HV5 to approx. 780HV5, and their value depends on the measurement location. The thinner coatings hardfaced in a single layer that have a higher proportion of core material components have a lower hardness. In the double-layer coatings, especially in their wider areas, hardness values near the journal surface are highest. In these areas, X-ray analysis of the chemical composition revealed alloying element contents similar to those found in the flux-cored wire with which the shafts were hardfaced. The hardnesses of the journal cores of the hardfaced shafts have different values and are significantly greater than the hardness of the tempered new shaft. The hardness of the journal core of the shaft hardfaced with a double-layer by double structural transformation is significantly lower than that of the journal of the shaft hardfaced with a single-layer.

## 4. Conclusions

- Worn surface layers of steel journals of turbocharger shafts of automotive engines (e.g., Peugeot 307-1.6 HDI and Laguna 1.9 DCI) can be subjected to the process of their rebuilding by arc hardfacing of hard coatings.
- Coatings applied to the journals of the remanufactured shafts are characterized by a fine-grained martensitic structure with fine spherical dispersive hard carbides containing M_23_C_6_-type chromium arranged in the matrix.
- As a result of surfacing with wire with powder cores with an increased content of 0.6% C and 5.4% Cr, coatings were obtained with a hardness twice as high as the hardness of the turbo-compressor shaft journal cores.
- Coating structure, with a hardness of almost twice that of the highly tempered martensite structure of new shafts, should, in turn, provide better abrasive wear resistance for these important structural components of turbochargers.

## Figures and Tables

**Table 1:** The result of the micro analysis of the chemical composition from points 1–4. The compactness of the elements % by weight.

No. of the Micro-Area	C	Si	Cr	Mn	Fe
1.	4.01 (16.15) *	-	4.18 (3.90) *	0.87 (0.77) *	balance
2.	4.01 (16.15) *	0.52 (0.89) *	3.86 (3.59) *	1.05 (0.93) *	balance
3.	5.28 (20.47) *	0.50 (0.83) *	3.82 (3.42) *	1.17 (0.99) *	balance
4.	4.48 (17.87) *	-	4.23 (3.90) *	1.11 (0.97) *	balance

* The compactness of the elements in atomic %.

**Table 2:** HV5 hardness values of coatings and journal cores of turbocharger shafts.

	Single-Layer Coating	Journal Core with Single-Layer Coating	Double-Layer Coating	Journal Core with Double-Layer Coating	New Shaft
Hardness, HV5	742, 749, 740, 745, 746	576, 575, 602, 580, 581	761, 772, 770, 773, 778	494, 423, 445, 450, 460	380, 384, 382, 380, 378
On average, HV5	745	580	770	455	380

## Data Availability

Not applicable.
